# Non-Invasive Ventilation as a Therapy Option for Acute Exacerbations of Chronic Obstructive Pulmonary Disease and Acute Cardiopulmonary Oedema in Emergency Medical Services

**DOI:** 10.3390/jcm11092504

**Published:** 2022-04-29

**Authors:** Felix C. F. Schmitt, Daniel Gruneberg, Niko R. E. Schneider, Jan-Ole Fögeling, Moritz Leucht, Felix Herth, Michael R. Preusch, Werner Schmidt, Christian Bopp, Thomas Bruckner, Markus A. Weigand, Stefan Hofer, Erik Popp

**Affiliations:** 1 Department of Anesthesiology, Heidelberg University Hospital, 69120 Heidelberg, Germany; daniel.gruneberg@med.uni-heidelberg.de (D.G.); niko.schneider@med.uni-heidelberg.de (N.R.E.S.); j.o.foegeling@gmail.com (J.-O.F.); moritzleucht@gmx.de (M.L.); markus.weigand@med.uni-heidelberg.de (M.A.W.); erik.popp@med.uni-heidelberg.de (E.P.); 2 Department of Pneumology and Critical Care Medicine, Thoraxklinik, University of Heidelberg, 69126 Heidelberg, Germany; felix.herth@med.uni-heidelberg.de; 3 Department of Internal Medicine III, Cardiology, University of Heidelberg, 69120 Heidelberg, Germany; michael.preusch@med.uni-heidelberg.de; 4 Department of Anesthesiology and Intensive Care Medicine, Thoraxklinik, University of Heidelberg, 69126 Heidelberg, Germany; werner.schmidt@med.uni-heidelberg.de; 5 Department of Anesthesiology and Intensive Care, GRN Hospital Schwetzingen, 68723 Schwetzingen, Germany; christian.bopp@grn2.de; 6 Institute of Medical Biometry and Informatics (IMBI), Heidelberg University, 69120 Heidelberg, Germany; bruckner@imbi.uni-heidelberg.de; 7 Department of Anesthesiology, Kaiserslautern Westpfalz Hospital, 67655 Kaiserslautern, Germany; shofer@westpfalz-klinikum.de

**Keywords:** prehospital non-invasive ventilation, acute respiratory insufficiency, acute cardiopulmonary oedema, acute exacerbated COPD, emergency medical service

## Abstract

In this observational prospective multicenter study conducted between October 2016 and October 2018, we tested the hypothesis that the use of prehospital non-invasive ventilation (phNIV) to treat patients with acute respiratory insufficiency (ARI) caused by severe acute exacerbations of chronic obstructive pulmonary disease (AECOPD) and acute cardiopulmonary oedema (ACPE) is effective, time-efficient and safe. The data were collected at four different physician response units and three admitting hospitals in a German EMS system. Patients with respiratory failure due to acute exacerbation of chronic obstructive pulmonary disease and acute cardiopulmonary oedema were enrolled. A total of 545 patients were eligible for the final analysis. Patients were treated with oxygen supplementation, non-invasive ventilation or invasive mechanical ventilation. The primary outcomes were defined as changes in the clinical parameters and the in-hospital course. The secondary outcomes included time efficiency, peri-interventional complications, treatment failure rate, and side-effects. Oxygenation under phNIV improved equally to endotracheal intubation (ETI), and more effectively in comparison to standard oxygen therapy (SOT) (paO_2_ SOT vs. non-invasive ventilation (NIV) vs. ETI: 82 mmHg vs. 125 mmHg vs. 135 mmHg, *p*-value SOT vs. NIV < 0.0001). In a matched subgroup analysis phNIV was accompanied by a reduced time of mechanical ventilation (phNIV: 1.8 d vs. ETI: 4.2 d) and a shortened length of stay at the intensive care unit (3.4 d vs. 5.8 d). The data support the hypothesis that the treatment of severe AECOPD/ACPE-induced ARI using prehospital NIV is effective, time efficient and safe. Compared to ETI, a matched comparison supports the hypothesis that prehospital implementation of NIV may provide benefits for an in-hospital course.

## 1. Introduction

Background: Non-invasive ventilation (NIV) is a well-established in-hospital therapy for acute respiratory insufficiency (ARI), but evidence is lacking with regard to its use in a prehospital setting. In particular, its prehospital use in comparison to ETI has not yet been characterized sufficiently. NIV is defined as mechanical ventilation by a non-invasive interface. Its therapeutic value has been evaluated in a variety of clinical trials. Favorable effects for in-hospital treatment have been shown for acute exacerbations of chronic obstructive pulmonary disease (AECOPD) and acute cardiopulmonary oedema (ACPE) [1,2,3,4]. NIV enhances oxygenation via positive end-expiratory pressure (PEEP) and reduces the work of breathing by adding pressure support. 

Importance: Data from in-hospital use show several benefits of NIV. At first NIV is shown to improve patients’ vital signs. Second, it has a beneficial influence on the clinical course. Mortality rates are reduced [5,6], the time of mechanical ventilation is shortened [1], and weaning success is increased [1]. Furthermore, NIV is performed without the need for general anesthesia and endotracheal intubation (ETI). Clinical trials suggest that, upon NIV indication, early implementation is beneficial [6,7,8,9,10]. The decrease in intubation rates is accompanied by a reduction in complications such as aspiration and ventilator-associated pneumonia [11,12,13]. While beneficial effects are shown in comparison to SOT in the prehospital setting and in comparison to SOT and ETI in in-hospital treatment, the availability of data is poor regarding the comparison to ETI in a prehospital setting. We try to expand the scope in a prospective multicenter study comparing phNIV to both SOT and ETI. 

Goals of this Investigation: The aim of this study is to evaluate the association of phNIV with clinical outcomes in patients with acute respiratory failure due to AECOPD and ACPE, in comparison to SOT and ETI. Furthermore, we examine the time efficiency and safety of phNIV.

## 2. Materials and Methods

Study design and setting: The study was conducted as an observational prospective multicenter study in the German EMS system of Heidelberg. Recruitment took place between October 2016 and October 2018. Patients were treated by four physician response units, two of which were located in an urban operational area, while the other two were located in a rural one. Thus, different tactical and medical circumstances were taken into account. Patients were admitted either to the University Clinic Heidelberg, a large over-regional university hospital providing maximum care; to the Thoraxklinik Heidelberg, a clinic specializing in thoracic diseases; or to the GRN Clinic Schwetzingen, a supplier of basic and customary care.

Selection of Participants: Patients were enrolled in the study if they were aged ≥18 years and presented with acute respiratory failure due to AECOPD or ACPE (final diagnosis obtained from in-hospital medical reports), and were treated by one of the participating physician response units and one of the participating hospitals. Furthermore, documentation of at least one blood gas analysis after admission was mandatory for inclusion.

Interventions: Respiratory failure was treated with one of the three following regimes: standard oxygen therapy (SOT), characterized by the administration of oxygen via face mask or nasal canula; non-invasive ventilation (NIV) encompassing the administration of positive end-expiratory pressure (PEEP) and pressure support or biphasic positive airway pressure ventilation (BIPAP) via non-invasive interface (face mask); or ETI, defined as endotracheal intubation with consecutive mandatory mechanical ventilation. For NIV and ETI, respirator settings were adjusted by the EMS physician according to individual patient needs. All patients received additional pharmacological therapy according to the current standard of care. Allocation to one of the treatment regimens was based on clinical decisions made by the EMS physicians. If reasonable clinical therapy was performed as a step-up approach, an initial SOT trial was performed first. In cases of further deterioration and failure of former therapy, treatment was escalated to NIV or ETI.

Measurements: For baseline characteristics, age, sex and comorbidities were obtained from EMS and hospital medical reports. Vital parameters comprising peripheral oxygen saturation, respiratory rate, heart rate, systolic blood pressure and Glasgow Coma Scale (GCS) were recorded at first EMS contact and at handover by the operating EMS physician. Blood gas analysis was conducted within the first hour after hospital admission. Any medication given during EMS treatment was documented. All physician response units were equipped with a survey containing questions about problems and complications during treatment, and details regarding respirator settings and user satisfaction. For clinical course analysis, the duration of mechanical ventilation, ICU length of stay, hospital length of stay, and mortality were extracted from the electronic medical reports.

Outcomes: Changes in vital signs between first EMS contact and handover, blood gas analysis within the first hour after hospital admission, and clinical course variables (time of mechanical ventilation, ICU length of stay, hospital length of stay and in-hospital mortality) were measured as primary outcomes. On-scene and transportation time, treatment failure rate, complications (alterations in hemodynamic parameters and vigilance, cardiac arrest) were defined as secondary outcomes.

Analysis: Data were obtained using digitalized EMS (NADOK, DATAPEC GmbH, Pliezhausen, Germany) and hospital medical reports. Statistical analyses were performed using SAS 9.4 (SAS Institute Inc., Cary, NC, USA). ANOVA and two-sided t-tests were used for continuous data and chi-squared tests for categorical data. Ordinal data were analyzed using a Kruskal–Wallis Test with a Dwass–Steel–Critchlow–Fligner post-hoc test for multiple comparison. Patients were matched for clinical course analysis via propensity score matching performed with SAS 9.4. Matching parameters were demographic characteristics (sex and age), the initial clinical parameters (peripheral oxygen saturation, respiratory rate, GCS-score) and the underlying pathology (AECOPD or ACPE). The caliper was set at 0.1. The Charlson Comorbidity Index and a modified APACHE II Score were used to ensure that comorbidities were balanced between groups. Modification of the APACHE II Score was performed via exclusion of temperature and paO_2_, since these parameters were not measured on-scene. Graphs were drawn in Prism 9 (GraphPad Software, San Diego, CA, USA). Statistical significance was defined as *p* < 0.05. 

The study was approved by the Ethics Committee of the Medical Faculty of Heidelberg (trial code no. S-203/2016) and is registered at the German clinical trials register (RKS00011041).

## 3. Results

### 3.1. Characteristics of Study Subjects

#### 3.1.1. Recruitment and Inclusion

**Figure 1:**
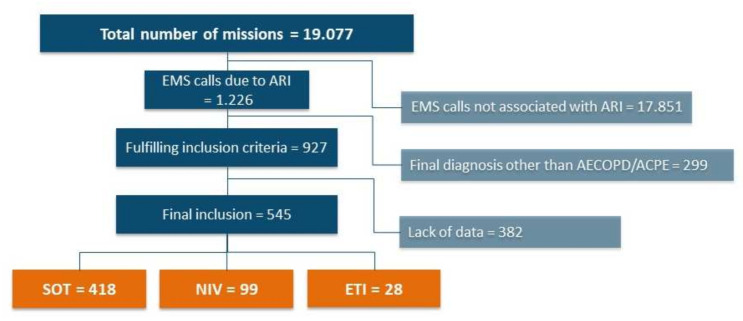
Data collection and inclusion. Flowchart of data collection process, data exclusion and allocation of patients to respective study groups, shown as number of patients. Abbreviations: EMS = emergency medical service, ARI = acute respiratory insufficiency, AECOPD = acute exacerbation of chronic obstructive pulmonary disease, ACPE = acute cardiopulmonary oedema, SOT = standard oxygen therapy, NIV = non-invasive ventilation, ETI = endotracheal intubation.

19,077 EMS missions were screened for this study and 927 patients fulfilled the inclusion criteria. A total of 382 patients had to be excluded due to missing data, leaving 545 patients eligible for the final analysis (Figure 1). Group allocation was based on the treatment modality at hospital arrival. Patients with an initial trial of standard oxygen therapy or NIV that showed treatment failure and a change of treatment were classified according to the treatment, which was performed at handover.

#### 3.1.2. Demographic Data

Regarding demographic data and pre-existing diseases, the baseline patient characteristics were comparable between all groups. On average, patients were 75 years old with a typical set of cardiopulmonary comorbidities. A detailed overview of the baseline characteristics and comorbidities divided by treatment group is provided in Table 1.

#### 3.1.3. Initial Clinical Data

At first EMS contact, patients who were assigned to ventilatory support showed higher respiratory rates and lower peripheral oxygen saturation before treatment. The lowest oxygen saturation was found in patients allocated to the ETI group (Table 2). The hemodynamic parameters were comparable in all groups. Prior to treatment, patients in the ETI group showed lower levels of vigilance compared to patients in the SOT and NIV groups. The median Glasgow Coma Scale (GCS) was lower in the ETI group than in the SOT and NIV groups.

### 3.2. Primary Outcomes

#### 3.2.1. Handover Clinical Data

**Figure 2:**
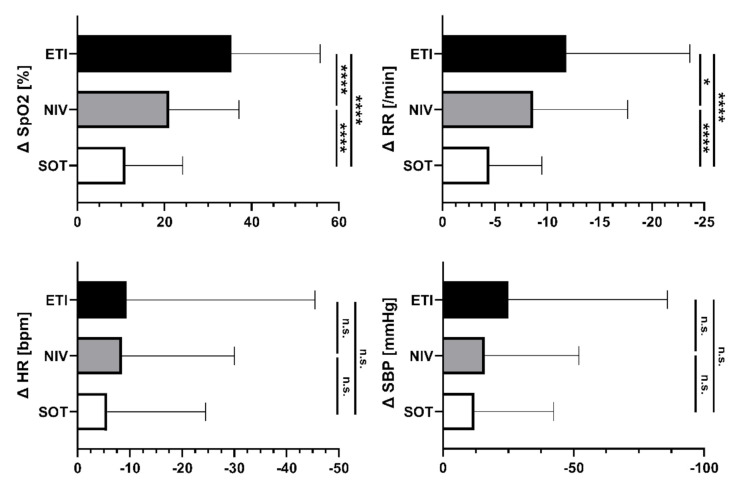
Absolute changes in respiratory and circulatory parameters at hospital admission in each study group. Data are presented as mean absolute change between first contact and hospital admission and standard deviation. A positive value on the *x*-axis shows an increase in the parameter and a negative value indicates a decrease; *p* > 0.05 = not significant (n.s.), *p* < 0.05 = *, *p* < 0.0001 = ****. Abbreviations: SpO_2_ = saturation of peripheral oxygen, RR = respiratory rate, HR = heart rate, SBP = systolic blood pressure, SOT = standard oxygen therapy, NIV = non-invasive ventilation, ETI = endotracheal intubation.

The clinical parameters improved during EMS treatment in all the study groups. At hospital admission, 89.2% of patients presented with peripheral oxygen saturation > 90% (89% under SOT, 94% under NIV and 79% under ETI). Improvements in oxygenation were accompanied by reduced respiratory drive, indicated by a reduction in respiratory rate (Table 2). This effect was more pronounced in the NIV and ETI groups than in the SOT group (Figure 2).

**Figure 3:**
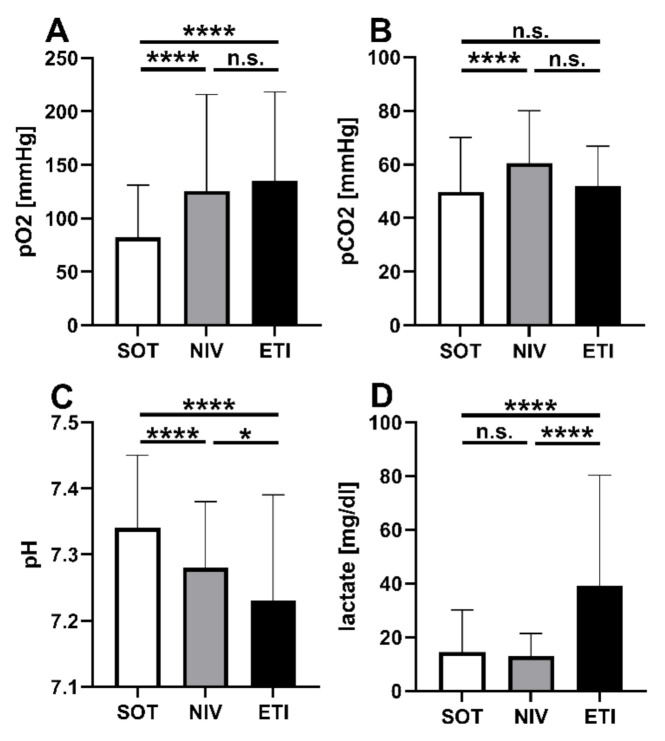
First blood gas analysis after hospital admission. Arterial or capillary blood gas analysis after hospital admission: (**A**) oxygenation stated as pO_2_ = partial pressure of O_2_; (**B**) elimination of CO_2_ stated as pCO_2_ = partial pressure of carbon dioxide; (**C**) acid-base status stated as pH value; (**D**) plasma lactate levels. Data are plotted as mean and standard deviation; *p* > 0.05 = not significant (n.s.), *p* < 0.05 = *, *p* < 0.0001 = ****. Abbreviations: SOT = standard oxygen therapy, NIV = non-invasive ventilation, ETI = endotracheal intubation.

Within the first hour after hospital admission, blood gases were taken. The results were in line with vital signs (Figure 3). The oxygen partial pressure in the NIV group was comparable to ETI (paO_2_ NIV vs. ETI = 126 mmHg vs. 135 mmHg; mean difference = 9 mmHg; 95% CI = −30–47; *p* = 0.67) and superior to SOT (paO_2_ = 126 mmHg vs. 82 mmHg, mean difference = 44 mmHg; 95% CI = 30–57; *p* < 0.001). Patients under NIV showed sufficient elimination of CO_2_. Even though initial CO_2_ values on-scene were not measured, carbon dioxide partial pressure did not differ significantly between NIV and ETI at handover (mean difference = 7 mmHg 95% CI = −15.5–0.7; *p* = 0.07).

**Figure 4:**
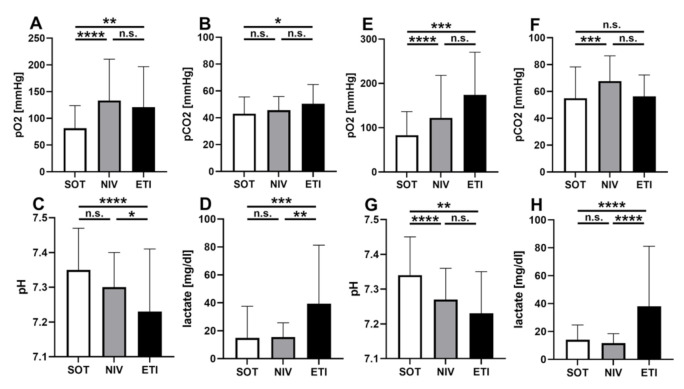
First blood gas analysis after hospital admission by underlying pathology. Arterial or capillary blood gas analysis after hospital admission from ACPE (**A**–**D**) and AECOPD (**E**–**H**) subgroups. (**A**,**E**): oxygenation stated as pO_2_ = partial pressure of oxygen; (**B**,**F**): elimination of CO_2_ stated as pCO_2_ = partial pressure of carbon dioxide; (**C**,**G**): acid-base status stated as pH value; (**D**,**H**): plasma lactate levels. Data are plotted as mean and standard deviation; *p* > 0.05 = not significant (n.s.), *p* < 0.05 = *, *p* < 0.01 = **, *p* < 0.001 = *** *p* < 0.0001 = ****. Abbreviations: SOT = standard oxygen therapy, NIV = non-invasive ventilation, ETI = endotracheal intubation.

A subgroup analysis in which blood gases were analyzed for ACPE and AECOPD separately revealed that phNIV provided sufficient oxygenation in both entities. The PaO_2_ values were superior to SOT and comparable to ETI in both subgroups. The elimination of CO_2_ in the ACPE group showed no significant differences between SOT and NIV; however, in the AECOPD group, CO_2_ values were higher in patients treated with NIV. The difference reached statistical significance in comparison to SOT, but not in comparison to ETI (Figure 4).

#### 3.2.2. Clinical Course

We further evaluated the association of phNIV with the clinical course in severe cases treated by either NIV or ETI, as this group of patients is particularly prone to complications. We performed propensity score matching to generate a subset of patients with comparable parameters for demographic data, initial vital signs and comorbidities, and analyzed their clinical outcomes depending on prehospital treatment. After matching, the groups did not differ in terms of initial vital signs (peripheral oxygen saturation, respiratory rate, heart rate, systolic blood pressure, Glasgow Coma Scale) and proportion of AECOPD/ACPE as the underlying pathology, or in their Charlson Comorbidity Scores and modified APACHE II scores as indicators of comorbidity and case severity. It was possible to match 13 subjects in the ETI group with 21 in the phNIV group. phNIV was related to a reduced timespan of mechanical ventilation (phNIV: 1.8 d vs. ETI: 4.2 d; mean difference = 2.4 d; 95% CI = 0.46–4.32; *p* = 0.03) and length of ICU stay in comparison to ETI (3.4 d vs. 5.8 d; mean difference = 2.4 d; 95% CI = 0.39–4.54; *p* = 0.02). Hospital length of stay did not differ significantly (phNIV: 6.8 d; ETI: 10.2 d; mean difference = 3.5 d; 95% CI = −3–10). In both groups, four cases of in-hospital mortality occurred. The mortality rates (22.2% under NIV vs. 30.8% under ETI; risk difference = 8.6%; 95% CI = −23–40) did not differ significantly between the groups.

### 3.3. Secondary Outcomes

#### 3.3.1. Hemodynamic Parameters, Vigilance and Sedation

At hospital admission, patients treated with NIV presented with more stable hemodynamics, lower lactate, less acidosis and less catecholamine use compared to the ETI group. While patients in the SOT and NIV groups showed signs of hemodynamic instability (SBP < 90 mmHg, and/or need for norepinephrine) in 8.1% and 5.1%, respectively; 89.3% of patients in the ETI group fulfilled this criterion. Mean norepinephrine use was significantly higher in the ETI group than in the other two groups (100 µg norepinephrine under ETI vs. 0 µg under SOT and NIV, *p* < 0.001). Furthermore, ETI was accompanied by deep sedation. While sedatives were also used under SOT and NIV, these patients did not show relevant reduction in vigilance. Sedation under SOT and NIV was performed predominantly with intravenous morphine. The mean morphine dose was 6.9 mg under NIV, compared to 3.0 mg under SOT.

#### 3.3.2. Time Efficiency

NIV was associated with shorter on-scene and transportation times in comparison to ETI. Time expenditures were comparable to SOT. Prehospital NIV took an additional 5.7 min on-scene compared to SOT (95% CI 3–8). However, in comparison to ETI, NIV was associated with a reduction in on-scene time (mean reduction 20 min; 95% CI = 15–26; *p* < 0.001). Transportation time was also slightly prolonged in the ETI group. All values for on-scene and transportation time are given in Table 3.

#### 3.3.3. Complication Rates

Failures in NIV therapy with the need to switch the therapeutic regime and cardiac arrest, and with the need for resuscitation, were monitored as peri-interventional complications. The overall complication rate was 2.6%. No aspiration of gastric contents was recognized in any of the groups. Failure in NIV treatment was documented in seven cases. In all of these cases, therapy was escalated to ETI. All NIV failures occurred in cases of severe ARI with an initial respiratory rate > 30/min and/or initial oxygen saturation of less than 80%. Cardiac arrest occurred in eight cases: five under SOT, one under NIV and two under ETI (Table 3). No fatalities were reported under prehospital therapy in any of the groups. A total of 23.5% of patients treated with SOT needed ventilatory support during their hospital stay.

## 4. Discussion

NIV is well known to be effective in the in-hospital treatment of ARI [6,14,15]. During the last two decades, evidence suggesting that NIV can be a suitable therapeutic option, even in a prehospital setting, has accumulated [8,10,16,17,18]. While several randomized controlled trials showed that phNIV is beneficial in comparison to SOT [8,10,19], data comparing phNIV to ETI are scarce. In this study, we showed that phNIV improves respiratory parameters during prehospital treatment and that there is an association with a favorable clinical course in comparison to ETI.

Our data showed that patients who underwent phNIV and patients who underwent ETI presented with comparable oxygenation. In terms of the elimination of CO_2_, it was found that patients under phNIV in ACPE presented with similar pCO_2_ values when compared to ETI. A trend towards higher pCO_2_ values under NIV in subjects suffering from AECPOD was detected. This fact underlines the need for end-tidal CO_2_ monitoring, especially in hypercapnic patients. For them, end-tidal CO_2_ monitoring may offer an opportunity for the timely detection of treatment failure. phNIV seems to be to be safe and time efficient, especially in comparison to ETI. 

That phNIV improves the clinical course in comparison to SOT has been shown by different groups before [8,10,16]. Data from the matched comparison generate the hypothesis that phNIV may shorten the time of mechanical ventilation and the ICU length-of-stay in comparison to ETI. These results go along with the improvement in vital signs. Thus, phNIV might be an effective treatment and could present a suitable addition to therapeutic concepts for certain patient groups presenting with severe ARI due to ACPE or AECOPD.

Despite the association of phNIV with a favorable clinical course, we were not able to show a difference in mortality rates between phNIV and other treatment regimens. At this point, our data are in line with previous trials [16,18,19].

Even though it was not possible to detect mortality differences in this study, the association of phNIV with improvements in vital signs and a favorable clinical course implies the conduction of future randomized controlled trials to determine the effectiveness of phNIV in comparison to ETI [20]. Therefore, care needs to be exercised in patient selection, since compliance with known indications and contraindications of NIV is crucial for successful treatment. The questions that need to be answered are whether principles from in-hospital treatment can be transferred to the prehospital setting, and whether an initial phNIV trial to avoid ETI—as is performed in the hospital—is justified in the field.

ETI is an invasive treatment with a relevant complication rate [21]. These complications may be of particular importance in a prehospital setting where personal and technical resources are limited. In particular, a critically ill patient is susceptible to these complications [21,22,23,24,25,26]. Thus, it may be of importance to perform treatment in a step-up approach, beginning with non-invasive strategies to limit the need for ETI, particularly in severe cases [27]. Our data support the hypothesis that phNIV can be suitable even in severe ARI, as it is associated with improvements in vital signs and clinical outcomes in the population of this observational study.

While SOT may be a sufficient treatment for less severe cases [20], previous studies demonstrated that phNIV improves vital signs more sufficiently compared to SOT [8,10,17,18]. An improvement in clinical parameters could be reached within 5 minutes of treatment [8]. In line with the existing literature, we showed that the improvement in oxygenation and respiratory rate under phNIV was more profound compared to SOT. However, the stabilization of patients’ vital signs cannot be used as a reliable surrogate for further clinical course [28,29]. While SOT improved vital signs in certain patients, a significant proportion of patients still needed ventilatory support throughout the clinical course [20]. In our study, approximately one out of four patients treated with SOT needed ventilatory support during their hospital stay. This may indicate that the usage of phNIV has not yet reached its full potential. More patients may benefit from the early implementation of ventilatory support in order to avoid further deterioration and worsening of respiratory exhaustion.

We revealed that the implementation of phNIV provided a significant benefit in terms of time expenditure compared to ETI. Therefore, phNIV may offer a secondary benefit for critically ill patients, by shortening the time until hospital admission and definite treatment. 

Prehospital NIV is reported to be safe and feasible [8,17,18]. In existing studies, failure rates for phNIV ranged from 3–11% and were lower compared to SOT [17,18]. In our study, there was an overall NIV failure rate of 6.7%, which corresponds with the existing literature. We showed that all the NIV failures occurred in cases of severe ARI with an initial respiratory rate > 30/min and/or initial oxygen saturation of less than 80%. No aspiration events were reported. The fact that secondary intubation was more common under NIV compared to SOT reflects the fact that more severe cases were treated with NIV. The number of cardiac arrests was lower in the NIV group compared to SOT and ETI. Patients treated with phNIV presented with more stable hemodynamics and less catecholamine use compared to ETI. These results correspond with findings from in-hospital treatment, which indicate that NIV could be performed in hemodynamically compromised patients and in cardiogenic shock [15,30]. Our data suggest that NIV can be performed safely in a prehospital setting in critically ill subjects. Consideration of phNIV in order to avoid ETI, therefore, seems justified, even in severe-ARI and hemodynamically compromised patients.

For decades now, NIV has been well established in the clinical setting. Nowadays, the expertise from clinical practice is transferred into prehospital care, with the use of phNIV continuously rising [31]. In less severe cases, NIV may act as a bridging therapy until medication begins to work [18]. In more severe cases, it may serve as a rescue strategy for therapy failure under SOT, and prevent ETI and ETI-linked complications [32,33]. Data from this observational study support the hypothesis that phNIV may lead to beneficial effects with regard to vital parameters and the clinical course in comparison to ETI. Nevertheless, the effectiveness of phNIV in comparison to ETI needs to be further evaluated in randomized controlled trials, in order to bring the results from this hypothesis-generating study to a cause–effect relationship. In addition, there needs to be an evaluation of whether the effectiveness of phNIV can be extended to further pathologies. Particularly in times of COVID-19, with overloaded healthcare and EMS systems, it is conceivable that phNIV represents a reasonable addition to the established therapeutic spectrum. The latest data indicate that NIV is feasible for COVID-19 disease and may reduce intubation rates in cases of COVID-19 as well [34,35].

Since this study is based on an observational design, it was not possible to control for group equality in the context of underlying pathophysiology and disease severity in the whole study population. Potential confounders were reduced by propensity score matching, but cannot be excluded entirely. As a side-effect of the matching, the sample size was significantly reduced which, of course, limits the explanatory power of the results obtained from the matched comparison. Furthermore, NIV is a procedure that requires a large amount of experience in implementation and a well-trained team. However, NIV was well established in all EMS units and admitting hospitals participating in this study. All the EMS physicians were experienced in the implementation and maintenance of NIV and ETI/invasive mechanical ventilation, so it can be assumed that an adequate level of expertise was provided in all the treatments. At this point, the heterogeneity of emergency medical services in an international context should also be acknowledged. While the system in Germany and other European countries is two-tiered, with paramedics and EMS physicians at the scene, this is not the case in other parts of the world such as most parts of the United States [36,37]. One has to be cautious to not generalize the results from a physician-based EMS system to a paramedic-based one. Nevertheless, there are data indicating that phNIV can be performed safely by emergency medical technicians [18]. Finally, data from blood gas analysis revealed that pCO_2_ under phNIV was slightly higher compared to other treatment regimes in the subgroup of AECOPD. It was not possible to tell whether this difference occurred due to the given treatment or whether it was pre-existing, as there was no possibility for CO_2_ monitoring on the scene. These results underline the need for end-tidal CO_2_ monitoring not only for ETI but also for phNIV. Because of the given study design, it is not possible to determine the causal effects between the different treatment modalities and the clinical course. This study was of an exploratory character, and should facilitate the randomized controlled trials that are needed to evaluate the potential benefits of the prehospital use of NIV in comparison to ETI.

## 5. Conclusions

In conclusion, our data reveal that phNIV improves respiratory parameters even in severe ARI caused by AECOPD and ACPE. phNIV was accompanied by a reduction in the duration of mechanical ventilation and ICU-LOS compared to ETI in a matched comparison. Prehospital use seemed to be safe and time-efficient, and complication rates were low. With regard to mortality rates, the group size was too small to draw solid conclusions. While previous work compared NIV to SOT, our study suggests that NIV may offer potential benefits in more severe cases, and that a prehospital NIV trial could be justified before intubation if known contraindications have been taken into account. We want to encourage randomized controlled trials comparing the prehospital use of NIV in comparison to ETI, to prove the benefits of phNIV in severe ARI based on a cause and effect relationship.

## Figures and Tables

**Table 1:** Demographic data and baseline characteristics.

	Total	SOT	NIV	ETI	*p*-Value
Sex (female/male), *n* (%)	227/318 (42/58)	174/244 (42/58)	41/58(41/59)	12/16(43/57)	*p* = 0.99
Age (years)	75.1 ± 10.7	75.5 ± 10.6	73.8 ± 11.3	74.1 ± 10.0	*p* = 0.28
LTOT	167 (30.6%)	126 (30.1%)	37 (37.4%)	4 (14.3%)	*p* = 0.06
OSAS	34 (6.2%)	25 (6.0%)	7 (7.1%)	2 (7.1%)	*p* = 0.97
Asthma	18 (3.3%)	12 (2.9%)	5 (5.0%)	1 (3.6%)	*p* = 0.83
Arterial hypertension	468 (85.9%)	357 (85.4%)	86 (86.9%)	25 (89.3%)	*p* = 0.95
s/p myocardial infarction	141 (25.9%)	114 (27.3%)	21 (21.2%)	6 (21.4%)	*p* = 0.42
PAOD	62 (11.4%)	51 (12.2%)	8 (8.1%)	3 (10.7%)	*p* = 0.51
Renal failure	134 (24.6%)	110 (26.3%)	18 (18.2%)	6 (21.4%)	*p* = 0.22

Demographic data and baseline characteristics. Data are presented as number of patients and percentage or mean ± standard deviation as appropriate. Abbreviations: SOT = standard oxygen therapy, NIV = non-invasive ventilation, ETI = endotracheal intubation, LTOT = long-term oxygen therapy, OSAS = obstructive sleep apnea syndrome, s/p = status post, PAOD = peripheral artery occlusive disease.

**Table 2:** Initial and handover clinical data.

Initial Clinical Data					
	Total	SOT	NIV	ETI	*p*-Value
Respiratory rate (/min)	24 ± 9	23 ± 7	29 ± 11	28 ± 11	SOT vs. NIV: *p* < 0.001 SOT vs. ETI: *p* = 0.002 NIV vs. ETI: *p* = 0.56
SpO_2_ (%)	81 ± 14	84 ± 12	76 ± 16	62 ± 19	SOT vs. NIV: *p* < 0.001 SOT vs. ETI: *p* < 0.001 NIV vs. ETI: *p* < 0.001
Heart rate (/min)	105 ± 28	103 ± 28	114 ± 23	110 ± 43	SOT vs. NIV: *p* < 0.001 SOT vs. ETI: *p* = 0.24 NIV vs. ETI: *p* = 0.47
SBP (mmHg)	149 ± 38	148 ± 36	156 ± 40	134 ± 53	SOT vs. NIV: *p* = 0.06 SOT vs. ETI: *p* = 0.08 NIV vs. ETI: *p* = 0.01
GCS	15 (15, 15)	15 (15, 15)	15 (13, 15)	12 (8, 14)	SOT vs. NIV: *p* < 0.001 SOT vs. ETI: *p* < 0.001 NIV vs. ETI: *p* = 0.001
Handover clinical data					
	Total	SOT	NIV	ETI	*p*-Value
Respiratory rate (/min)	19 ± 6	18 ± 6	21 ± 6	16 ± 3	SOT vs. NIV: *p* < 0.001 SOT vs. ETI: *p* = 0.03 NIV vs. ETI: *p* < 0.001
SpO_2_ (%)	95 ± 6	95 ± 7	97 ± 3	96 ± 5	SOT vs. NIV: *p* = 0.01 SOT vs. ETI: *p* = 0.53 NIV vs. ETI: *p* = 0.52
Heart rate (/min)	99 ± 20	98 ± 21	105 ± 17	98 ± 22	SOT vs. NIV: *p* < 0.001 SOT vs. ETI: *p* = 0.93 NIV vs. ETI: *p* = 0.11
SBP (mmHg)	135 ± 24	135 ± 23	140 ± 24	112 ± 22	SOT vs. NIV: *p* = 0.06 SOT vs. ETI: *p* < 0.001 NIV vs. ETI: *p* < 0.001
GCS	15 (14, 15)	15 (15, 15)	14 (12, 15)	3 (3, 3)	SOT vs. NIV: *p* < 0.001 SOT vs. ETI: *p* < 0.001 NIV vs. ETI: *p* < 0.001

Initial and handover clinical data. Data are presented as median (upper and lower quartile) for GCS and mean ± standard deviation for all other values. Abbreviations: SOT = standard oxygen therapy, NIV = non-invasive ventilation, ETI = endotracheal intubation, SBP = systolic blood pressure, SpO_2_ = saturation of peripheral oxygen, GCS = Glasgow Coma Scale.

**Table 3:** Time consumption and cardiac arrests.

	Total	SOT	NIV	ETI	*p* Value
On-scene time (min)	29 ± 13	27 ± 11	32 ± 12	53 ± 15	SOT vs. NIV: *p* < 0.001 SOT vs. ETI: *p* < 0.001 NIV vs. ETI: *p* < 0.001
Transportation time (min)	17 ± 9	16 ± 8	16 ± 8	22 ±13	SOT vs. NIV: *p* = 0.82 SOT vs. ETI: *p* < 0.001 NIV vs. ETI: *p* = 0.001
CPR (*n*)	8 (1.5%)	5 (1.2%)	1 (0.9%)	2 (13.3%)	SOT vs. NIV: *p* = 0.83 SOT vs. ETI: *p* = 0.17 NIV vs. ETI: *p* = 0.51

Time consumption and cardiac arrests. Data are presented as mean ± standard deviation for on-scene and transportation time and the number and percentage of patients in which CPR was performed. Abbreviations: SOT = standard oxygen therapy, NIV = non-invasive ventilation, ETI = endotracheal intubation, CPR = cardiopulmonary resuscitation.

## Data Availability

Generated during the study.
